# Single‐Cell Transcriptomics Uncovers Core Signature for Regulating Mitochondrial Homeostasis During Testicular Ageing

**DOI:** 10.1111/cpr.13797

**Published:** 2024-12-27

**Authors:** Weijie Xu, Qiuru Huang, Yujuan Qi, Qingqing Hu, Cong Shen, Xia Chen, Jiaxin Li, Qiushi Xia, Ziyue Pan, Yi Zhang, Guoqing Han, Jingqi Huang, Yiheng Liu, Ziyu Cao, Ying Zheng, Bo Zheng, Zhifeng Gu, Jun Yu, Chi Sun

**Affiliations:** ^1^ Department of Geriatrics, Affiliated Hospital of Nantong University Medical School of Nantong University Nantong China; ^2^ Institute of Reproductive Medicine, Medical School of Nantong University Nantong University Nantong China; ^3^ Clinical Center of Reproductive Medicine, Xuzhou Central Hospital Xuzhou Clinical School of Xuzhou Medical University Xuzhou China; ^4^ State Key Laboratory of Reproductive Medicine and Offspring Health, Center for Reproduction and Genetics, The Affiliated Suzhou Hospital of Nanjing Medical University, Suzhou Municipal Hospital Gusu School of Nanjing Medical University Suzhou China; ^5^ Department of Histology and Embryology, School of Medicine Yangzhou University Yangzhou China; ^6^ Department of Rheumatology, Affiliated Hospital of Nantong University Nantong University Nantong China

**Keywords:** *Drosophila*, mitochondrial function, oxidative phosphorylation, spermatogenic cells, testicular ageing

## Abstract

Testicular ageing is accompanied by a series of morphological changes, while the features of mitochondrial dysfunction remain largely unknown. Herein, we observed a range of age‐related modifications in testicular morphology and spermatogenic cells, and conducted single‐cell RNA sequencing on young and old testes in *Drosophila*. Pseudotime trajectory revealed significant changes in germline subpopulations during ageing. Our examination unveiled that genes showing bias in spermatids exhibited higher dN/dS than those in GSCs_Spermatogonia. Genes biased towards young GSCs_Spermatogonia displayed higher dN/dS than those in old GSCs_Spermatogonia. Interestingly, genes biased towards young spermatids demonstrated lower dN/dS in contrast to those in old spermatids, revealing the complexity of evolutionary adaptations during ageing. Furthermore, mitochondria associated events, including oxidative phosphorylation, TCA cycle and pyruvate metabolism, were significantly enriched in germline subpopulations. Specifically, mitochondrial function was significantly impaired during the process of testicular ageing, concurrently emphasising the role of several key nuclear genome‐encoded mitochondrial regulatory genes, such as Hsp60B, fzo, Tim17b1 and mRpL12. Our data offer insights into testicular homeostasis regulated by mitochondrial function during the ageing process.

## Graphical abstract

We observed a range of age‐related modifications in testicular morphology and spermatogenic cells during ageing, and conducted scRNA‐seq on testes, establishing a foundational comprehension of mitochondrial dysfunction as a basis for testicular ageing. By shedding light on spermatogenic cells during testicular ageing, we identify novel targets associated with mitochondrial biological occurrences.

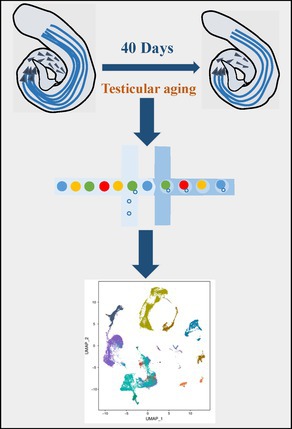

## 1 Background

Ageing is an inherent process characterised by physiological alterations in various bodily systems and has been linked to a decline in reproductive capabilities [1]. Testicular ageing, categorised as one kind of organisational ageing, is recognised as an intricate biological progression resulting in compromised functionality within the testes [2]. Research has consistently demonstrated that advancing male age is correlated with prolonged time to conception and reduced pregnancy rates [3]. The prevalence of these occurrences has sparked concerns regarding the influence of ageing and premature senescence on fertility and reproductive risks. As crucial reproductive structures, mounting evidence suggests that the rise in male age markedly impairs spermatogenesis, sperm functionality, fertilisation, pregnancy outcomes and the health of offspring [4, 5, 6].

The progressive deterioration of testicular function as men age has sparked scientific curiosity in the realm of testicular ageing. Previous studies utilising single‐cell RNA sequencing (scRNA‐seq) have delineated the transcriptomic profile of testes in young and aged mice, unveiling numerous differentially expressed genes (DEGs) associated with ageing [7]. While these findings offer an initial insight into the transcriptional features and disparities between young and aged testes, further comprehensive research involving diverse species is imperative to pinpoint critical biological occurrences and regulatory elements in testicular ageing.

Oxidative stress stands out as a primary mechanism contributing to reproductive alterations during testicular ageing [8]. However, genes biased towards age and essential factors linked to mitochondrial function within spermatogenic cells across the ageing continuum remain incompletely understood. The highly conserved spermatogenesis and short life cycle render *Drosophila melanogaster* a valuable model for investigating testicular ageing [9, 10, 11]. In this study, we systematically assessed ageing characteristics during spermatogenesis and established a transcriptional regulatory network for testicular ageing at single‐cell resolution in *Drosophila* testes. Our study primarily delved into mitochondrial‐associated biological occurrences to unearth novel mitochondrial targets within germ cells amid testicular ageing, potentially offering fresh perspectives on age‐related testicular function.

## 2 Results

### 2.1 Characterisation of Testicular Aging in *Drosophila*

**FIGURE 1:**
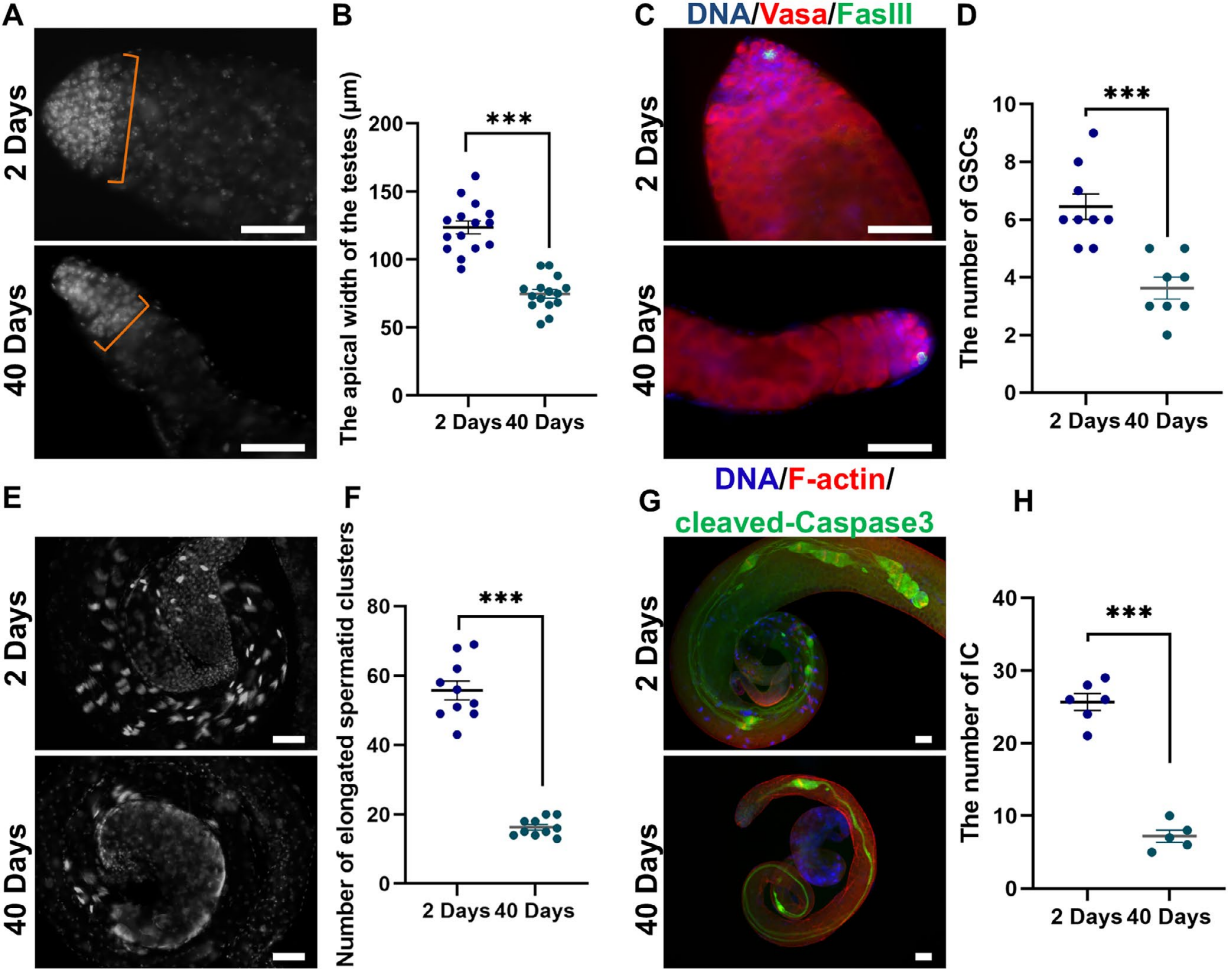
Testicular phenotypic analysis during the process of ageing. (A) DNA (grey) staining in 2‐days and 40‐days testicular samples. (B) Measurement of the apical width in 2‐days (*n* = 15) and 40‐days (*n* = 15) *Drosophila* testes. (C) Immunostaining of Vasa (red) and FasIII (green) in 2‐days and 40‐days testicular samples. (D) Quantification of GSCs at the apex of 2‐days (*n* = 9) and 40‐days (*n* = 8) testes. (E) Observation of elongated spermatid clusters at the tail of 2‐days and 40‐days testes. (F) The number of elongated spermatid clusters in 2‐days (*n* = 10) and 40‐days (*n* = 10) testes. (G) Stainings of cleaved‐Caspase3 (green) and F‐actin (red) in 2‐days and 40‐days testes. (H) Quantification of ICs in 2‐days (*n* = 6) and 40‐days (*n* = 5) testes. DNA staining was performed using Hoechst 33342 (blue). Statistical significance denoted as ***p < 0.001. Scale bar: 50 μm.

To shed light on the transformations occurring in testicular ageing, we initiated an investigation into the morphological characteristics of testes at 2‐days and 40‐days in *Drosophila*. In this inquiry, we observed a gradual decrease in the breadth of testicular tissue with advancing age (Figure 1A). Our statistical results of the apical width of the testes revealed a significant decrease in apical width at 40‐days compared with the 2‐days specimens (Figure 1B). Subsequently, we thoroughly delineated the alterations in germ cells throughout the entire process of spermatogenesis during testicular ageing. In contrast to the 2‐days samples, there was a reduction in the population of GSCs (germ cells neighbouring the hub cells) in the 40‐days testes, indicating a potential compromise in the spermatogenic process as ageing progresses (Figure 1C,D). To further investigate the potential effects on elongated spermatids, we proceeded to quantify the clusters of elongated spermatids at the posterior end of the testes. Our observations revealed a notable decrease in the clusters of elongated spermatids in *Drosophila* testes at 40‐days as opposed to 2‐days (Figure 1E,F). Following this, we employed staining with Phalloidin (F‐actin) and cleaved‐Caspase‐3 to examine the individualisation complex (IC), cystic bulges (CBs) and waste bags (WBs) during spermatid elongation. As expected, the 40‐days testes displayed pronounced reductions in the quantities of IC, CBs and WBs at the base of the spermatid cyst (Figure 1G,H). Collectively, these findings provide an initial insight into the modifications in testicular morphology and spermatogenic cells during testicular ageing in *Drosophila*.

### 2.2 Single‐Cell Transcriptome Profile During Testicular Ageing in *Drosophila*

**FIGURE 2:**
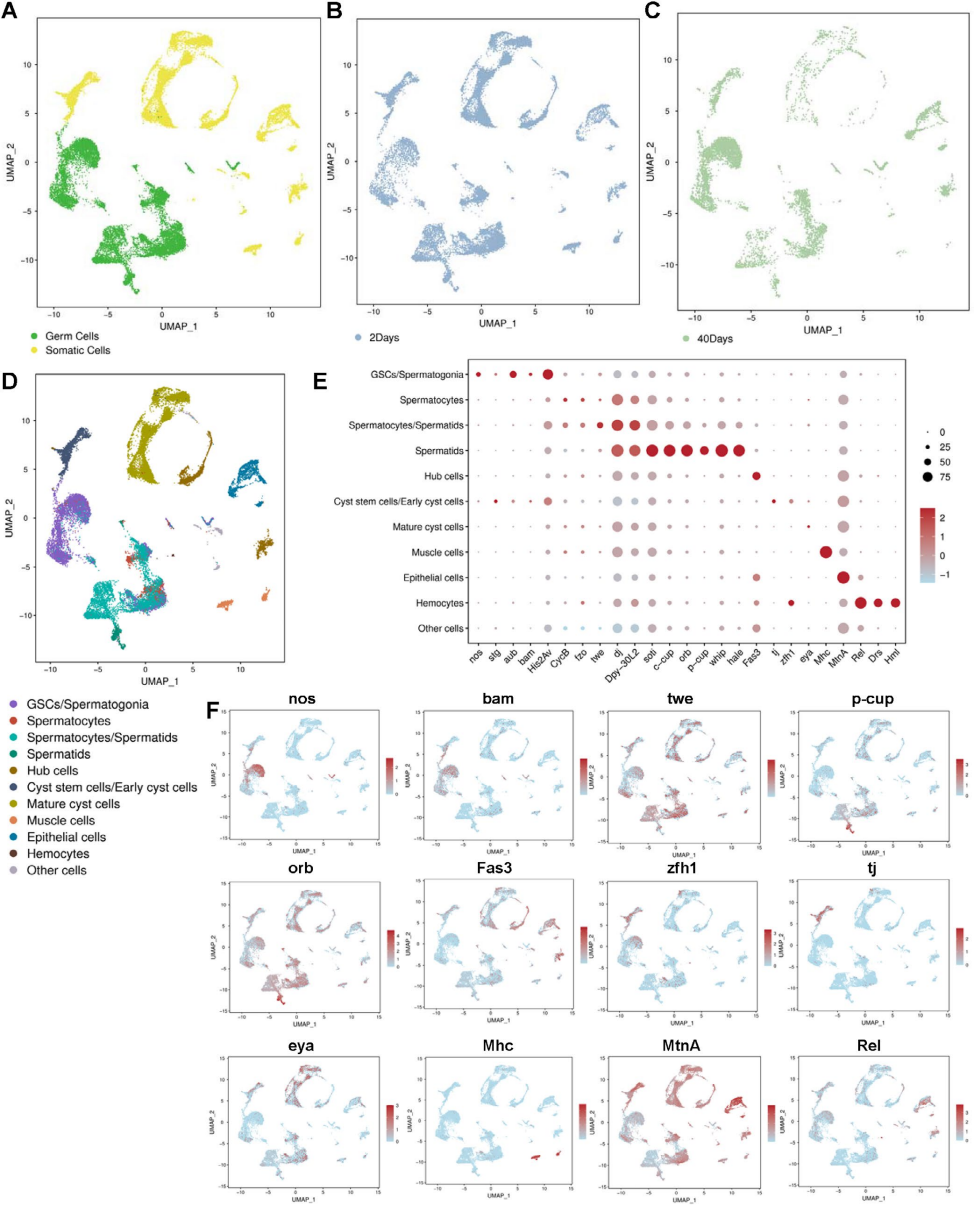
Generation of scRNA‐seq profiles in ageing‐related testes. (A) UMAP visualisation of testicular cell populations, distinguished between germ cells and somatic cells. (B) UMAP visualisation of testicular cells in 2‐days aged testes. (C) UMAP visualisation of testicular cells in 40‐days aged testes. (D) UMAP visualisation of testicular cells highlighting distinct cell clusters. (E) Dotplot visualisation depicting representative marker genes in each testicular cell cluster. The dot size corresponds to the proportion of cells expressing the gene within each cluster, while colour intensity indicates the average normalised expression level. (F) UMAP visualisations of representative marker genes.

To enhance comprehension of the molecular regulatory network associated with testicular ageing at the single‐cell level, we performed scRNA‐seq on cells isolated from freshly collected testes at 2 and 40 days post‐eclosion. After filtration, we retained 27,639 cells of exceptional quality for subsequent analyses, with an average values of 4602 median unique molecular identifiers (UMIs) and 1424 median genes per cell. The dimensionality of the gene/cell expression matrix was reduced to two primary axes and visualised using uniform manifold approximation and projection (UMAP) in Seurat 7 [12]. By grouping similar cells into clusters, UMAP plots depicted germ cells and somatic cells (Figure 2A). Furthermore, it was noted that several cell clusters showed a significant decrease in cell numbers with progressive testicular ageing (Figure 2B,C; 2‐days vs. 40‐days). Following our analysis, we proceeded to assign putative cell types to each cluster based on the expression patterns of established marker genes [13]. This classification resulted in the identification of 11 distinct cell populations, encompassing nearly all testicular cell types in *Drosophila* (Figure 2D,E). Additionally, we designated a cluster as an unannotated cellular sub‐cluster labelled ‘Other cells’. This comprehensive categorisation based on marker gene expression patterns allowed us to delineate the diverse cellular populations present in the *Drosophila* testes.

Multiple cell clusters were notably distinguished by a variety of marker genes, with their expression patterns elucidated through UMAP visualisations (Figure 2F). In the context of spermatogenesis in *Drosophila*, distinct stages of germ cells were clearly identifiable based on different marker genes [12, 14]. Specifically, the nanos (nos) gene, known as a marker for germline stem cells (GSCs), displayed prominent expression during the early phases of germ cell development [15]. The bag of marbles (bam) gene, responsible for a critical differentiation factor, exhibited specific expression during spermatogonia transit‐amplifying divisions (TA‐divisions), playing a crucial role in the transition from proliferation to meiotic differentiation [16]. Cyclin B (CycB) expression was biased towards mature spermatocytes [17]. Additionally, fuzzy onions (fzo) mRNA accumulated in spermatocytes and early spermatids, while genes such as calcutta cup (c‐cup), presidents‐cup (p‐cup), don juan (dj) and oo18 RNA‐binding protein (orb) were notably expressed in spermatids [17, 18, 19, 20]. Moreover, a heatmap illustrated the top 5 highly expressed genes in each cell cluster of the *Drosophila* testis (Figure S1), thereby enhancing the importance of marker genes in our investigation.

### 2.3 Quality Control of Cellular Sub‐Clusters in *Drosophila* Testes

**FIGURE 3:**
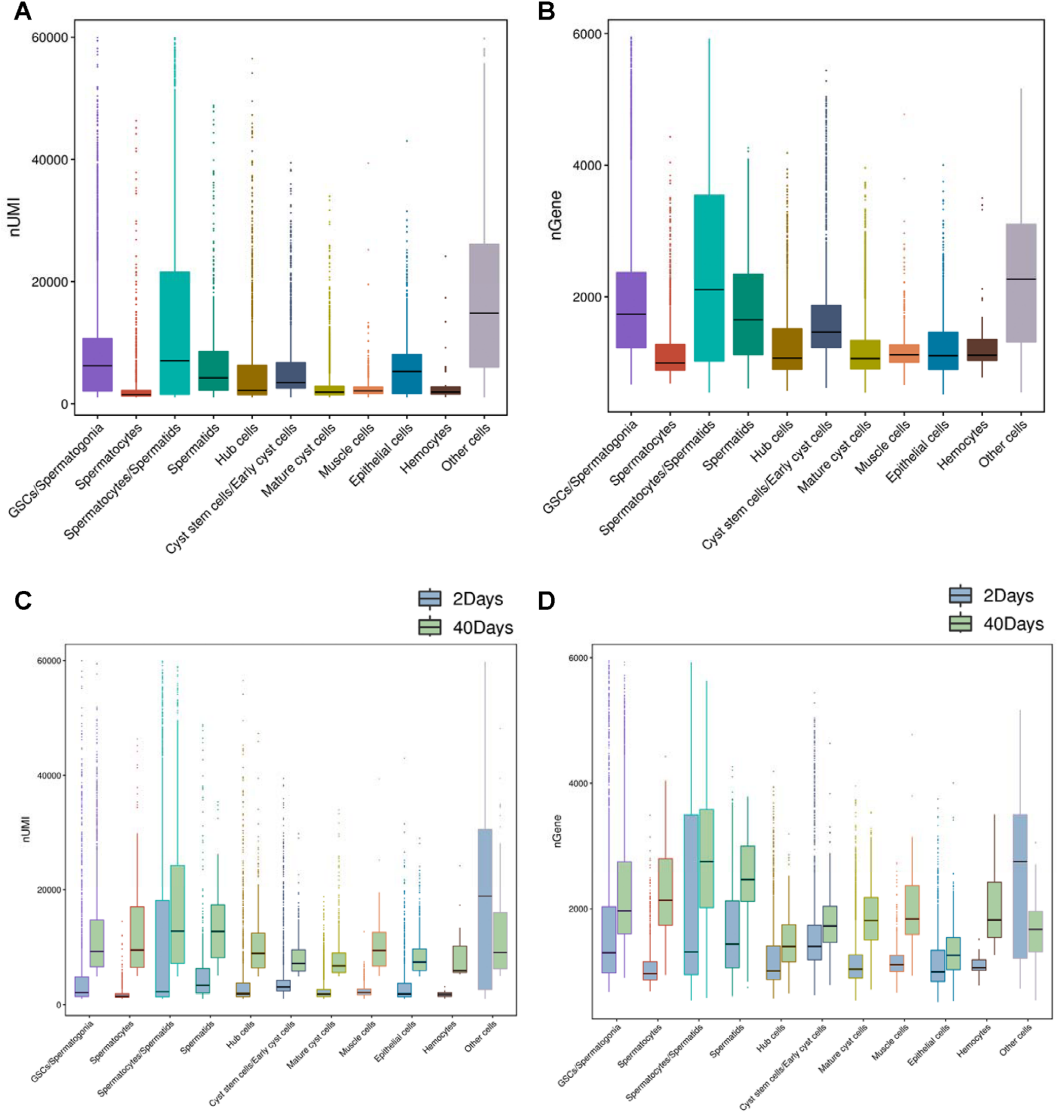
Quality control of scRNA‐seq data for age‐related testes. (A) The number of UMI (nUMI) for testicular cellular populations. (B) The number of gene (nGene) for testicular cellular populations. (C) Comparison of nUMI among 11 cell clusters in 2‐days and 40‐days aged testes. (D) Comparison of nGene among 11 cell clusters in 2‐days and 40‐days aged testes.

In our investigation, we quantified RNA contents and gene numbers using nUMIs and nGenes within each testicular sub‐cluster. Notably, our results revealed that several spermatogenic cell populations, including GSCs/Spermatogonia, Spermatocytes/Spermatids and Spermatids, demonstrated comparatively higher levels of nUMIs and nGenes in contrast to other germline populations in *Drosophila* testes (Figure 3A,B). It is noteworthy that both nUMIs and nGenes of all identified spermatogenic populations exhibited significant increases during testicular ageing, as illustrated in Figure 3C,D. These findings allowed us to present an initial overview of the trends in RNA content and gene numbers across different germline sub‐clusters within *Drosophila* testes.

### 2.4 Pseudotime Trajectory of Germ Cell Complexity During Testicular Aging

**FIGURE 4:**
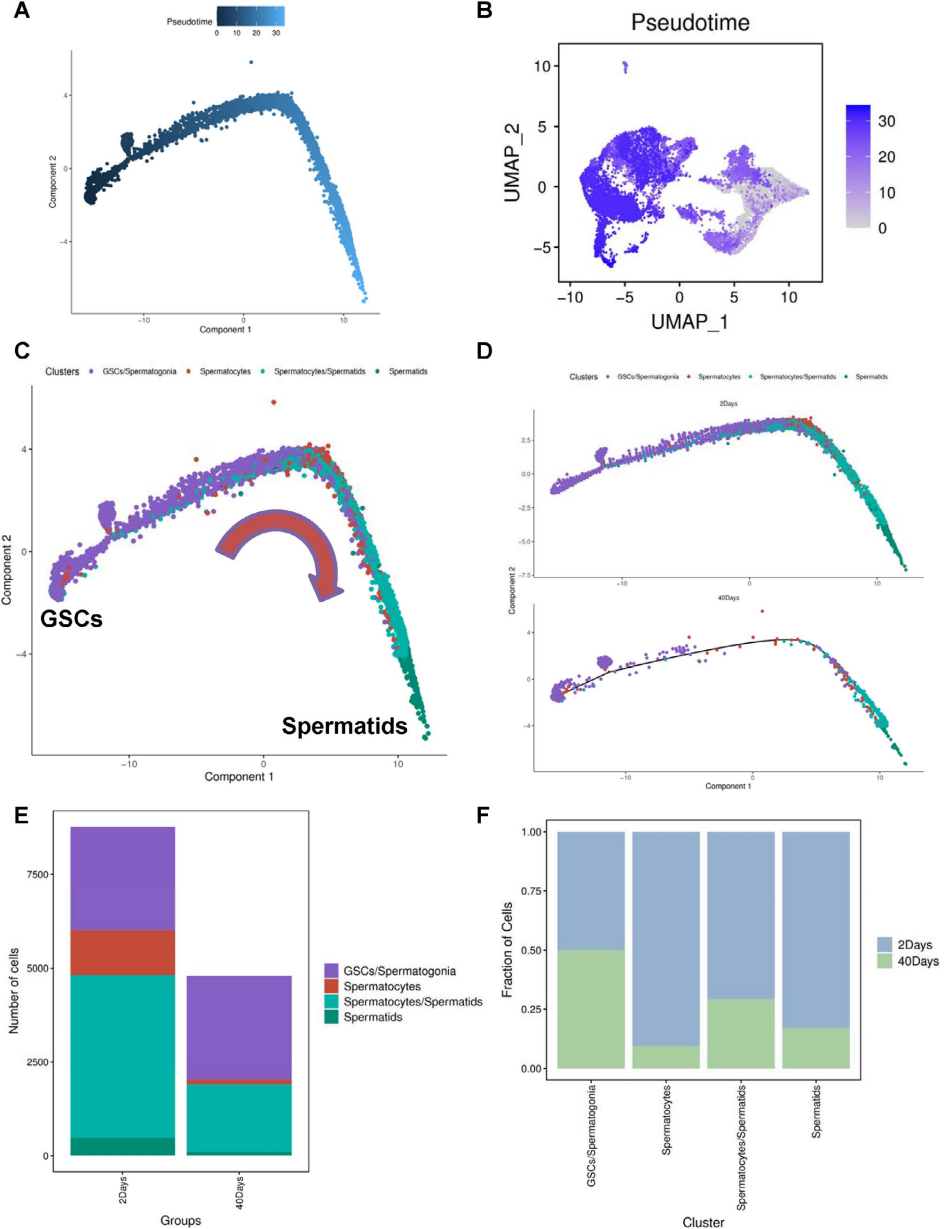
Pseudotime trajectory analysis of spermatogenic cells during the ageing process in testes. (A) Visualisation of pseudotime trajectory plot depicting germ cell populations in *Drosophila* testes. (B) UMAP visualisation of pseudotime trajectory analysis, with cells colour‐coded according to pseudotime. (C) Pseudotime trajectory analysis of germ cells, distinguished by various cell populations. (D) Pseudotime trajectory analysis of germ cells in 2‐days and 40‐days aged testes, distinguished by various cell populations. (E) Statistical analysis of cell counts for germline populations in 2‐days and 40‐days aged testes. (F) Statistical analysis of cell ratio for germline populations in 2‐days and 40‐days aged testes.

Our subsequent focus centred on exploring the intricacy of germ cells during testicular ageing. We reanalysed the data, concentrating on pseudotime trajectory analysis specifically within the germ cell clusters, as illustrated in Figure 4A,B. The pseudotime trajectory analysis unveiled distinct germline subpopulations aligning with the differentiation patterns observed during spermatogenesis (Figure 4C). Notably, the pseudotime trajectory analysis utilising density and RidgePlot in 2‐day‐old and 40‐day‐old testes highlighted significant shifts in germline subpopulations throughout testicular ageing, as depicted in Figure S2A,B. Furthermore, a comparative examination between the germ cell populations in 2‐day‐old and 40‐day‐old testes revealed a marked decrease in the majority of germ cells during testicular ageing (Figure 4D–F and Figure S2C). These discoveries offer valuable insights into the dynamic alterations in germline subpopulations during testicular ageing, providing illumination on the evolution of spermatogenesis over time.

### 2.5 dN/dS Trends of Age‐Biased Genes During Testicular TE Aging

**FIGURE 5:**
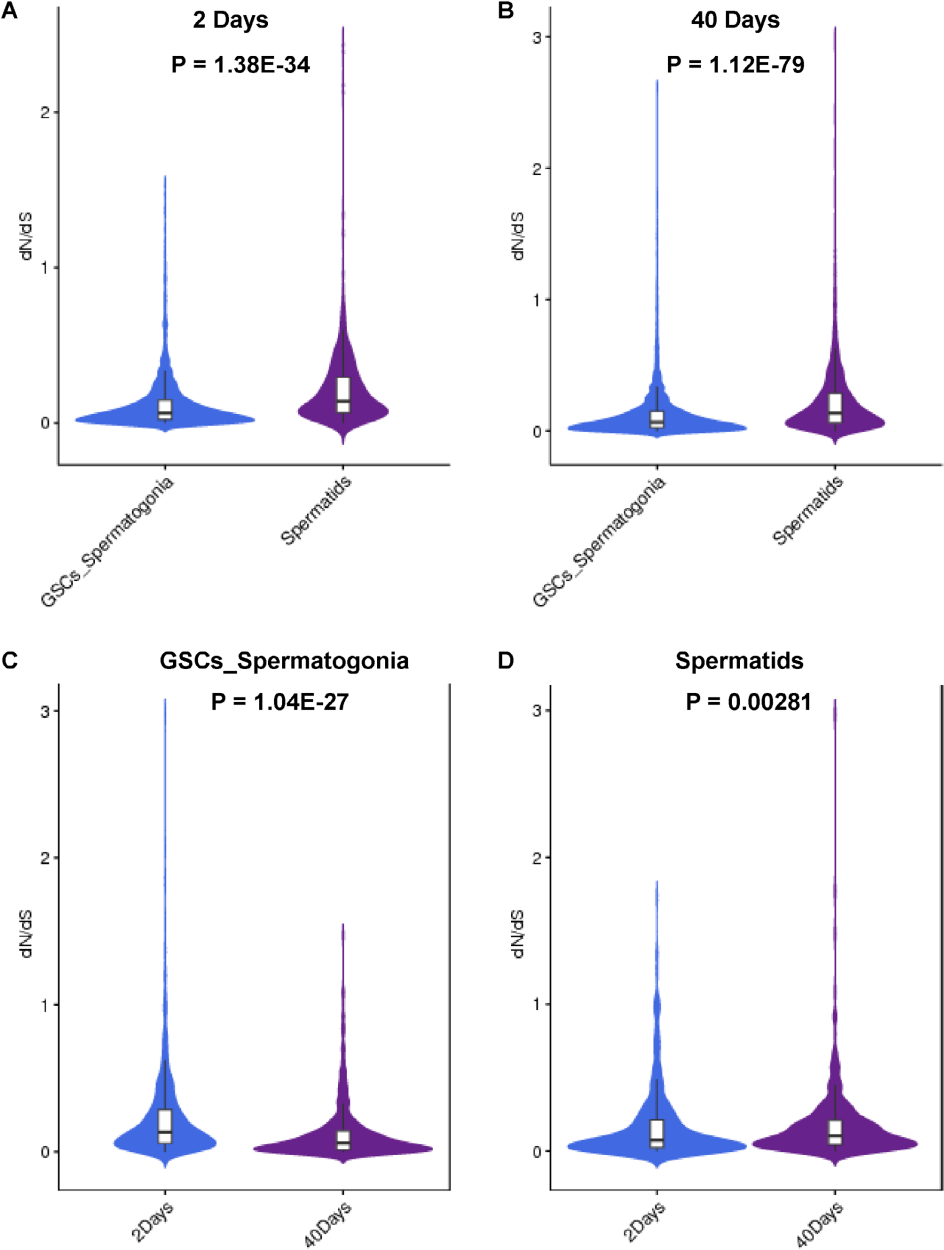
Distinctive alterations of age‐biased genes in germ cell populations during the ageing process. (A) Comparison of dN/dS values between GSCs_Spermatogonia and spermatids clusters in 2‐days aged testes. (B) Comparison of dN/dS values between GSCs_Spermatogonia and spermatids clusters in 40‐days aged testes. (C) dN/dS values comparison between 2‐days and 40‐days aged testes in GSCs_Spermatogonia populations. (D) dN/dS values comparison between 2‐days and 40‐days aged testes in spermatids populations. The dN/dS values are sourced from flyDIVaS. Genes with a dS value of 0 (or lacking a numerical value, or both dN and dS values equal to 0, or exhibiting an anomalous dN/dS value) were excluded.

We next conducted a comparative analysis of the non‐synonymous to synonymous substitutions ratio (dN/dS) values for age‐biased genes within early and late stages of germline subpopulations via flyDIVaS30 [21]. Prior studies have indicated that genes biased towards the late stage of spermatogenesis exhibit higher dN/dS values compared to those biased towards the early stage, supporting the idea that the late stage of spermatogenesis drives evolutionary innovation [22]. Our investigation also revealed that genes exhibiting bias in the spermatids cluster displayed higher dN/dS values in comparison to those in the GSCs_Spermatogonia cluster within both 2‐day and 40‐day testes (Figure 5A,B). Additionally, Witt et al. demonstrated that genes biased towards young germ cells (2‐days) exhibited higher dN/dS values than those biased towards aged germ cells (25‐days) [22]. Notably, our findings indicated that genes biased towards young GSCs_Spermatogonia cellular populations (2‐days) displayed higher dN/dS values than genes biased towards 40‐days aged GSCs_Spermatogonia cellular populations (Figure 5C). Interestingly, we observed that genes biased towards young spermatids clusters (2‐days) demonstrated lower dN/dS values in contrast to genes biased towards 40‐days aged spermatids clusters (Figure 5D), thereby unveiling the complex nature of evolutionary innovation during the late stage of spermatogenesis in the context of testicular ageing.

### 2.6 Identification of Novel Mitochondrial Associated Targets During Testicular Aging

**FIGURE 6:**
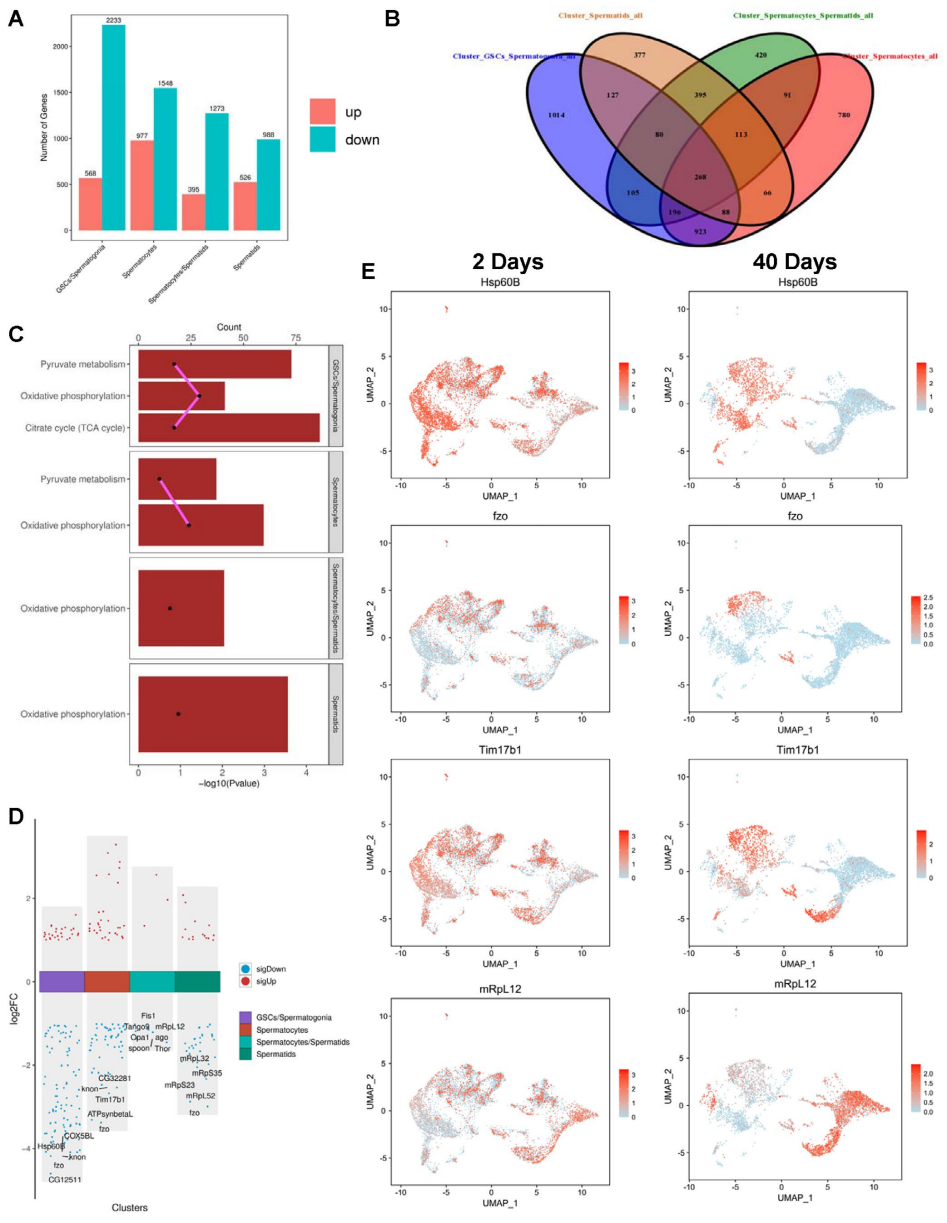
Fundamental transcriptional control during testicular ageing. (A) The quantity of DEGs in GSCs_Spermatogonia, Spermatocytes, Spermatocytes/Spermatids and spermatids clusters, respectively. (B) Venn diagram illustrating the shared DEGs among germ cell populations. (C) Barplot representations of KEGG enrichment related to mitochondrial‐associated processes in each germ cell population. (D) Display of down‐regulated and up‐regulated DEGs in each germ cell population. Noteworthy DEGs were emphasised in the respective graphs. (E) UMAP views of representative DEGs (Hsp60B, fzo, Tim17b1 and mRpL12) in germline cell populations aged 2‐days and 40‐days.

To explore the transcriptional regulation of germline subpopulations during testicular ageing at single‐cell resolution, we conducted an analysis of DEGs, revealing 568 up‐regulated and 2233 down‐regulated DEGs in the GSCs/Spermatogonia cluster, 977 up‐regulated and 1548 down‐regulated DEGs in the Spermatocytes cluster, 395 up‐regulated and 1273 down‐regulated DEGs in the Spermatocytes/Spermatids cluster, and 526 up‐regulated and 988 down‐regulated DEGs in the Spermatids cluster (Figure 6A). Subsequently, we utilised Venn diagram representations to identify 268 common DEGs across all four germline subpopulations (Figure 6B), with a predominant presence of commonly down‐regulated DEGs (Figure S3A,B).

Moreover, through Kyoto Encyclopedia of Genes and Genomes (KEGG) and gene ontology (GO) analyses, a series of mitochondrial related events, such as oxidative phosphorylation, tricarboxylic acid (TCA) cycle, pyruvate metabolism and mitochondria, were involved in the DEGs (2‐days vs. 40‐days) of germline subpopulations during testicular ageing (Figure 6C and Figure S3C,D). Notably, oxidative phosphorylation emerged as a prominently enriched process crucial across all four germline subclusters (Figure 6C). Within the DEGs identified during testicular germline ageing, we pinpointed several genes exhibiting markedly reduced expression levels in specific germ cell subpopulations (Figure 6D). The distinct stages of germline subclusters were visually depicted through UMAP representations (Figure S3E). Particularly, we illustrated UMAP views of Heat shock protein 60B (Hsp60B), fzo, Tim17b1 and mitochondrial ribosomal protein L12 (mRpL12) in the 2‐day and 40‐day testicular germline populations (Figure 6E). Furthermore, these factors (Hsp60B, fzo, Tim17b1 and mRpL12), encoded by the nuclear genome, were all detected in testicular cells via fluorescence in situ hybridisation (FISH) assay, and were found with significant decreases in 40‐day‐old testes when compared with 2‐day‐old testes (Figure S4), shedding light on the pivotal roles of these candidate targets in the context of testicular germline ageing.

### 2.7 Mitochondrial Function Was Impaired During Testicular Ageing

**FIGURE 7:**
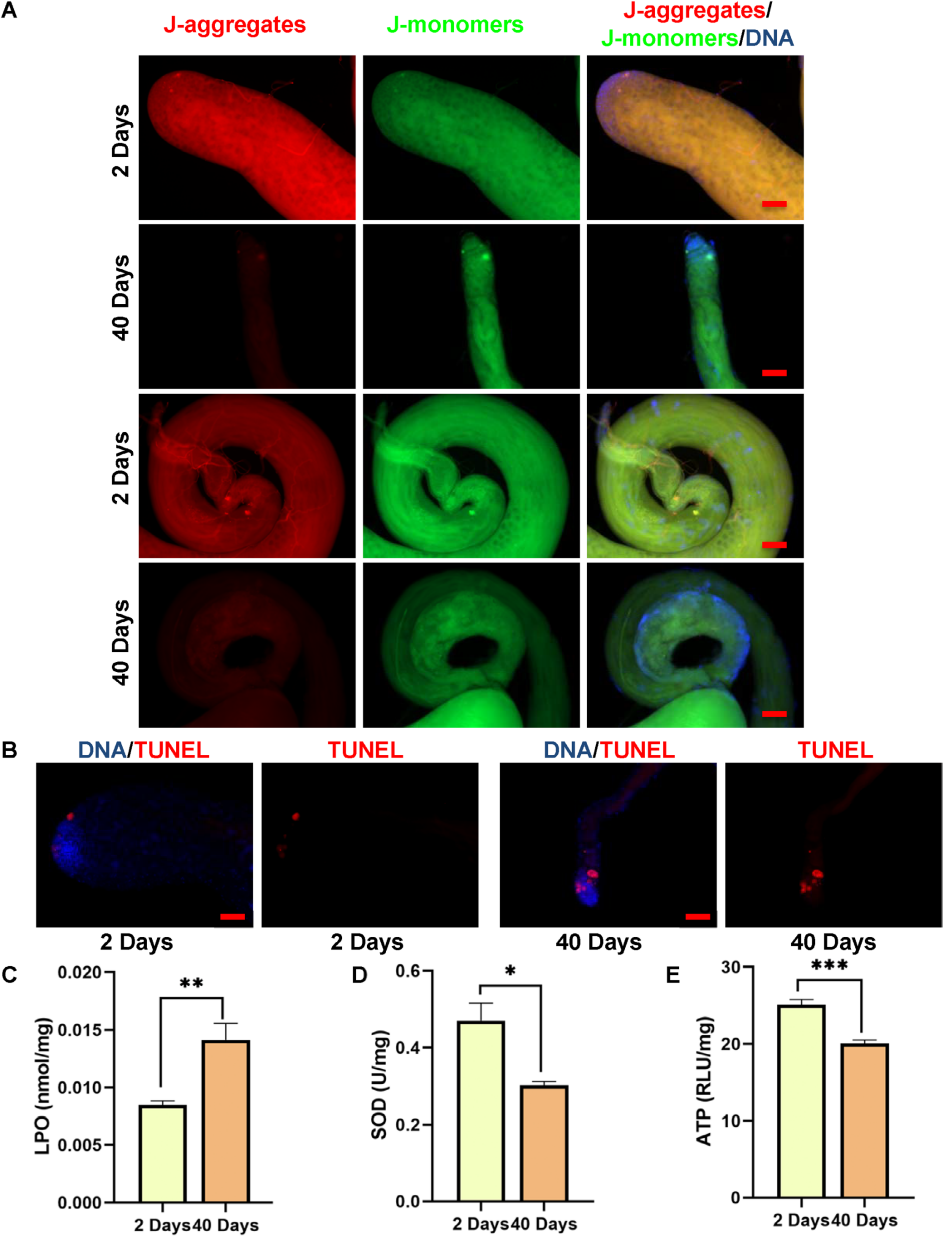
Assessment of mitochondrial function during testicular ageing. (A) JC‐1 staining of the apex and tail regions of testes at 2‐days and 40‐days. (B) TUNEL staining of testes aged 2‐days and 40‐days. (C) LPO level in testes aged 2‐days and 40‐days. (D) SOD activity in testes aged 2‐days and 40‐days. (E) ATP level in testes aged 2‐days and 40‐days. *p < 0.05, **p < 0.01, ***p < 0.001. DNA was counterstained with Hoechst33342. Scale bar: 50 μm.

Since mitochondrial dysfunction represented a hallmark of the ageing process [1], we proceeded to investigate the mitochondrial‐associated biological phenomena in *Drosophila* testes aged 2‐Ddays and 40‐days. JC‐1, a membrane‐permeable dye sensitive to mitochondrial membrane potential [13, 23], was employed to assess mitochondrial functionality. Our analysis unveiled with diminished mitochondrial membrane potential in both the apex and tail regions of the 40‐day‐old testes when compared to 2‐day‐old counterparts (Figure 7A). In contrast to the 2‐day‐old testes, we also observed a marked increase in TUNEL‐positive cells in the 40‐day‐old testes (Figure 7B). Subsequently, we investigated the impact of oxidative stress during testicular ageing. Noticeably, we observed an elevation in LPO level, and decreases in SOD activity and ATP level in 40‐day‐old testes in comparison to 2‐day‐old testes (Figure 7C–E). Furthermore, mitochondrial complex I‐V activities were decreased in 40‐day‐old testes in comparison to 2‐day‐old testes, including NADH dehydrogenase, Succinate‐Coenzyme Q reductase, CoQ‐Cytochrome C reductase, Cytochrome C oxidase and F1‐F0 ATP synthase (Figure S5). These findings collectively suggest a severe impairment in mitochondrial function during the process of testicular ageing.

## 3 Discussion

The negative reproductive consequences of increased parental age are often attributed primarily to females, with scant investigation into the effects of paternal age advancement. Nevertheless, the testes, a crucial reproductive organ, manifest age‐related impairment, characterised by reduced sperm quality and male fertility typically beginning around the age range of 40–45 years in humans [3]. Although age‐related alterations in the testes can impact the state of spermatogenic cells [24], the regulatory processes dictating age‐related testicular dysfunction remain somewhat enigmatic.

Spermatogenesis represents an intricate and dynamic cellular differentiation process crucial to male reproduction [16, 25, 26]. Hermann et al. previously documented single‐cell transcriptomes for individual spermatogenic cells from immature and adult male mice as well as adult men, elucidating gene expression alterations during spermatogenesis and identifying unique gene expression signatures for distinct spermatogenic cell types [27]. While the expression profiles of mammalian germ cells have been delineated, the regulatory mechanisms governing several key cell populations from testicular tissues during ageing, particularly those involving highly conserved molecular pathways across species, remained incompletely understood. Recent scRNA‐seq analysis of human testes afflicted with late‐onset hypogonadism unveiled dysregulated Sertoli cells with perturbed phagolysosomal and autolysosomal functions as crucial metabolic regulators during testicular ageing [28]. Another investigation illustrated the local cellular composition reflecting inflammaging in the initial segment of the mouse epididymis during the ageing process [29]. Our prior research also unveiled the diversity of terminal epithelium populations in the testes, akin to the role of the epididymis in mammals, in orchestrating the final phase of sperm release through various methodologies in *Drosophila* [30]. In our current study, we systematically assessed the phenotypic attributes of testicular ageing utilising a *Drosophila* model, with a specific focus on exploring the molecular characteristics of spermatogenic cells at the single‐cell resolution level. Our investigation primarily concentrated on elucidating the impact of mitochondrial function on spermatogenic cells themselves, highlighting the significant role of oxidative stress during testicular ageing.

Mitochondria play pivotal roles in energy production, cellular state maintenance and mitochondrial metabolic balance through diverse mechanisms such as ATP synthase, oxidative phosphorylation, TCA cycle and mitochondrial electron transport chain [31, 32, 33, 34]. Mitochondrial function is influenced by genetic, environmental and oxidative factors [35, 36]. A prior investigation revealed that exposure to cadmium leads to osteoporosis by triggering cellular senescence, linked to the activation of the NF‐κB pathway and mitochondrial dysfunction [37]. Recently, evidence also showed that copper overload led to mitochondrial dysfunction by Cuproptosis and further accelerated lncRNA deficiency‐induced testicular senescence, revealing an interaction between environmental and genetic factors [38]. Several studies have reported tissue‐specific alterations in mitochondrial function and gene expression in mammalian model, linking changes in both nuclear and mitochondrial genetic components. For example, research on buffalo tissues has shown differential expression of nuclear‐derived mitochondrial succinate dehydrogenase genes, highlighting variability in mitochondrial function across tissues [PMID: 39425877] [39]. Similarly, studies on tissue heterogeneity in mitochondrial activity, biogenesis and mitochondrial protein gene expression demonstrate high variability across tissues [PMID: 37140692] [40]. Recent study revealed significant tissue‐specific differences in nuclear genome‐encoded OXPHOS complex I genes in female buffalo [PMID: 38878239] [41], and differential expression of ATP synthase genes [PMID: 38305843] [42]. Our data also confirmed that mitochondrial complex I‐V activities in the mitochondrial electron transport chains were crucial during testicular ageing. Previous study demonstrated that the deficiency of ND‐42, a subunit of the complex I protein in the mitochondrial electron transport chain, resulted in the impairments of mitochondrial derivatives by influencing the mitochondrial membrane potential and mitochondria encoded genes [13]. Leveraging the benefits of scRNA‐seq, we also identified several core targets encoded by nuclear genome, which likely governed mitochondrial function during the ageing process in the testes. The study has highlighted fzo as a critical factor in spermatid differentiation, encoding a transmembrane GTPase crucial for mitochondrial fusion and male infertility [43]. While fzo mRNA expression commenced in early spermatocytes, Fzo protein localisation occurred in the mitochondrial derivative of spermatids [17]. The knon gene localised to spermatid mitochondria, encoding a remarkably large testis‐specific paralog of ATP synthase subunit d, crucial for the internal structure of the Nebenkern and its subsequent disassembly and elongation [31]. Hsp60B, predicted to encode the conserved Hsp60 family chaperones in *Drosophila*, exhibited the specific expression solely in the testes and is indispensable for spermatid individualisation [44]. COX5BL, implicated in mitochondrial electron transport, participated in mitochondrial respiratory chain complex IV [45]. While the role of COX5BL in testicular function remained obscure, the mitochondrial electron transport chain has been shown to regulate spermatogenesis and male fertility [13], hinting at potential functions of COX5BL in *Drosophila* testes. Building upon this evidence, our research corroborated that these DEGs related to mitochondria might serve as crucial regulatory elements during testicular ageing.

Despite being widely used, scRNA‐seq has its constraints, such as cell heterogeneity, technical sensitivity and noise [42]. To identify cell heterogeneity within potentially complex cell populations, a wide range of computational applications have been devised [46, 47]. Utilising a threshold on expression levels has been commonly employed to eliminate genes with low expression, effectively addressing technical noise in data analysis [48].

By leveraging flyDIVaS database, the dN/dS ratio was employed to evaluate the impact of *Drosophila* divergence and selection: genes with low dN/dS ratios demonstrated significant protein conservation across species, while genes with high dN/dS values indicated rapid positive selection for amino acid fixation [21]. Various models of dN/dS evolution could offer valuable insights into the variability of sites within proteins, distinguishing between evolutionarily stable and conserved sites across species and those undergoing alterations due to positive selection or other evolutionary pressures [49, 50]. A prior study revealed that post‐meiosis biased genes displayed elevated dN/dS ratios compared with pre‐meiosis biased genes [22], aligning with our own findings. This emphasised the importance of the late stage of spermatogenesis in propelling evolutionary innovation. Interestingly, Witt et al. noted that genes biased in the 2‐days aged spermatid cluster exhibited higher dN/dS values than those biased in the 25‐days aged spermatid cluster [22]. Conversely, our research unveiled that genes biased in the 2‐days aged spermatid cluster displayed lower dN/dS values than those biased in the 40‐days aged spermatid cluster. This disparity underscores the intricate nature of evolutionary adaptations in the advanced stages of spermatogenic cell populations during testicular ageing.

In conclusion, our research establishes a foundational comprehension of mitochondrial dysfunction as a basis for testicular ageing. By shedding light on spermatogenic cells during the ageing process in the testes, we identify novel targets associated with common mitochondrial biological occurrences. The ongoing challenge of testicular ageing remains a significant issue. A thorough understanding of these complexities is essential for healthcare professionals to effectively counsel patients considering delaying parenthood for societal reasons or individuals seeking fertility treatments. By concentrating on these biological mechanisms and implementing strategies to promote testicular health, it is possible to mitigate age‐related alterations in the testes, thereby preserving reproductive function and ensuring sustained testicular vitality.

## 4 Materials and Methods

### 4.1 Fly Strains and Culture

w^1118^ line was used for in vivo experiments. With a relative humidity of 40%–60% and the same photoperiod as outside, all flies were raised at 25°C and replicated in vials containing standard cornmeal molasses agar media.

### 4.2 Preparation of Single‐Cell Suspension and scRNA Sequencing Library

The 40‐day‐old testes were dissected in cold phosphate‐buffered saline (PBS; HyClone, Logan, UT, USA), promptly transferring them into ice‐cold GEXSCOPE Tissue Preservation Solution (Singleron Biotechnologies, Cheshire, CT, USA). The testes underwent triple rinsing in Hanks Balanced Salt Solution (HBSS), digestion in 2 mL of GEXSCOPE Tissue Dissociation Solution (Singleron Biotechnologies) within a Singleron PythoN Automated Tissue Dissociation System for 15 min at 28°C, followed by centrifugation for 5 min at 500*g* and suspension in PBS. Trypan blue (Sigma‐Aldrich, St. Louis, MO, USA) was utilised for staining the samples, and their viability was assessed under a microscope.

The Singleron Matrix Single Cell Processing System facilitated the loading of single‐cell suspensions (1 × 105 cells/mL in PBS) into microfluidic devices. Subsequently, the GEXSCOPE Single Cell RNA Library Kit was adhered for the construction of scRNA‐seq library. Initially, the single‐cell suspension was introduced into the microchip to enable the partitioning of individual cells into distinct wells. Cell barcoding beads were then introduced into the microchip cells and underwent washing. Following this, cell lysis occurred using 100 μL of single‐cell lysis buffer in each well, with mRNA capture lasting 20 min at room temperature. The mRNA‐carrying beads were isolated from the microchip for reverse transcription and cDNA amplification to form the libraries. Subsequently, the cDNAs underwent purification, size selection and pooling before sequencing on an Illumina Novaseq 6000 (San Diego, CA, USA) to generate 150 bp paired end reads.

### 4.3 scRNA‐Seq Data Analysis

The raw data from the 2‐days aged testes were retrieved from our previous study [13]. An internal pipeline was utilised to process the raw reads of the 2‐days and 40‐days aged testes to generate gene expression profiles. Initially, reads lacking poly‐T tails were filtered out, and UMIs were isolated for each cell barcode. Subsequently, reads sharing the same cell barcode were grouped together based on gene and UMI information to ascertain the UMI count per gene in each cell. Subsequent analysis involved utilising UMI count tables for individual cellular barcodes. Cells exhibiting a high percentage of mitochondrial genes (> 25%) or an unusually high UMI count (> 60,000) were excluded. Additionally, cells with gene counts below 510 or exceeding 6000 were also removed from the analysis.

The Seurat software was utilised for clustering analysis and cell type identification [51, 52]. Individual cell‐by‐gene matrices for each sample were imported into Seurat version 3.1.1 for subsequent analysis [52]. Cell clusters were visualised using uniform manifold approximation and projection (UMAP). DEGs with log_2_ (FC) > 1 and a *p* value < 0.05 were considered significant. The clusterProfiler tool was employed for gene ontology (GO) and Kyoto Encyclopaedia of genes and genomes (KEGG) analyses of the DEGs to elucidate their biological functions or relevant pathways. Monocle 2 (version 2.10.1) was used for single cell trajectory analysis utilising gene expression data and cell matrices to reduce the research dimensionality to two dimensions and to order the cells [53, 54].

### 4.4 Immunofluorescence Staining

About 10 pairs of testes at 2‐ and 40‐days were dissected in 1× PBS, fixed in 4% paraformaldehyde (PFA) for 30 min, washed three times using 1% PBS‐Triton X‐100 (PBST), and incubated for 30 min in 5% bovine serum albumin (BSA). Primary antibodies were diluted in 5% BSA and testes were incubated at 25°C for 1 h, and then washed three times with 0.3% PBST. Secondary antibodies conjugated with A488, Cy3 or A647 (Jackson ImmunoResearch Laboratories, West Grove, PA, USA) were diluted with 5% BSA at 1:400, and incubated with the testes samples for 1 h at 25°C in the dark. Following three washes with 0.3% PBST, testes were stained for 5 min using Hoechst 33342 (1:800, C0031, Solarbio, Beijing, China) before finalising. The primary antibodies used in this study included: rabbit anti‐Vasa (a gift from Prof. Chao Tong, 1:2000); mouse anti‐Fasciclin III (FasIII) (DSHB, 1:50); and rabbit anti‐cleaved Caspase‐3 (Cell Signalling Technology, 1:200). Images were captured on Zeiss confocal microscope (Carl Zeiss, Oberkochen, Germany) and processed using Adobe Photoshop CS5 software (Adobe, San Jose, CA, USA).

### 4.5 IC Staining and JC‐1 Assays

The structure of IC in testes was stained using Alexa Fluor Plus555 Phalloidin (1:50; A30106, Invitrogen, Waltham, MA, USA). JC‐1 (C2006, Beyotime, Shanghai, China) was used as a mitochondrial membrane potential marker. Briefly, approximately 10 pairs of fly testes at 2‐ and 40‐days for each group were dissected in 1× PBS and incubated with related working solutions according to the manufacturer's instructions. Testes were then washed three times with 1× PBS. Images were captured on Zeiss confocal microscope (Carl Zeiss, Oberkochen, Germany) and processed using Adobe Photoshop CS5 software (Adobe, San Jose, CA, USA).

### 4.6 Cell Death Assay

One‐Step TdT‐mediated dUTP Nick‐End Labeling (TUNEL) Apoptosis Assay Kit (#C1090, Beyotime) was utilised under the manufacturer's instructions to identify cell death by using dUTP labelled fluorescent probe under the catalysis of TdT enzyme. For each group, approximately 10 pairs of testes at 2‐ and 40‐days were washed three times with 1× phosphate buffered saline (PBS) after 20 min of fixation in 4% paraformaldehyde (PFA). In the dark, a mixture of 5 L of TdT enzyme and 45 L of fluorescent labelling solution was created. After washing three times in 1× PBS and being exposed to the combination for 1 h at 28°C in the dark, testes were stained with Hoechst 33342 (1:800, C0031, Solarbio, Beijing, China).

### 4.7 Oxidative Stress Assays

Oxidative stress was assessed by superoxide dismutase (SOD; S311, Dojindo Molecular Technologies), and lipid peroxidation (LPO; BC5245, Solarbio). Approximately 50 pairs of testes at 2‐ and 40‐days were respectively collected and put into ice‐cold saline for homogenate with an ultrasonic homogeniser, and then centrifuged at 12,000*g* for 10 min. The supernatants of tissue were obtained for the measurements of SOD and LPO contents according to the manufacturer's instructions.

### 4.8 The Measurement of ATP Level

The ATP levels were assessed utilising an ATP assay (S0026, Beyotime, Shanghai, China). About 50 pairs of *Drosophila* testicular tissues at 2‐ and 40‐days were separately dissected in 1× PBS, and placed in 200 μL of lysis buffer. Following complete lysis, the sample underwent centrifugation at 12,000*g* for 5 min at 4°C, with retention of the supernatant. Subsequently, 100 μL of ATP detection working solution was introduced into the detection well and left to incubate for 5 min at room temperature. Thereafter, 20 μL of each sample was swiftly added to the detection well and thoroughly mixed. The relative ATP levels were measured using a microplate reader (BioTek Instruments Inc., Friedrichshall, Germany).

### 4.9 Fluorescence In Situ Hybridisation

According to the manufacturer's protocol, FISH was performed with the FISH kit (Ribo Bio Technology Co. Ltd., Guangzhou, China). Testes at 2‐ and 40‐days were initially fixed using 4% paraformaldehyde for a duration of 30 min at room temperature. Subsequently, they were permeabilised in a solution containing PBS with 1% Triton X‐100 on ice for another 30 min. Next, the testes were treated with pre‐hybridisation buffer at 37°C for 30 min, followed by hybridisation with a 20 μM Cy3‐labelled RNA FISH probe mix in a humid chamber at 37°C overnight. All the FISH probe mix, including Hsp60B, fzo, Tim17b1 and mRpL12, was synthesised by Ribo Bio Technology Co. Ltd. Next day, the testes were washed three times in 4 × SSC containing 0.1% Tween‐20 for 5 min at 42°C followed by a 5‐min wash at 42°C in 2 × SSC, and then a final 5‐min wash at 42°Cin 1 × SSC. After hybridisation, testes were stained with Hoechst‐33342 (Solarbio, 1.0 mg/mL) for 5 min before mounting.

### 4.10 Detection of Mitochondrial Respiratory Chain Complex Activity

Mitochondrial respiratory chain complex activity was measured by Mitochondrial complex I/NADH dehydrogenase Activity Assay Kit (BC0515, Solarbio), Mitochondrial complex II/Succinate‐Coenzyme Q reductase Activity Assay Kit (BC3230, Solarbio), Mitochondrial complex III/CoQ‐Cytochrome C reductase Activity Assay Kit (BC3245, Solarbio), Mitochondrial Complex IV/Cytochrome C oxidase Activity Assay Kit (BC0945, Solarbio) and Mitochondrial Complex V/F1‐F0 ATP synthase Activity Assay Kit (BC1445, Solarbio). According to the manufacturer's instructions, approximately 50 pairs of testes at 2‐ and 40‐days were respectively collected and immediately put into ice‐cold saline for homogenate with an ultrasonic homogeniser. After centrifugation at 600*g* for 10 min at 4°C, the supernatants of the samples were transferred to fresh centrifuge tubes and centrifuged at 11,000*g* for 15 min at 4°C. The supernatants obtained were cytosolic extracts utilised for assessing the leakage of mitochondrial complexes I‐V, whereas the precipitates were isolated cellular mitochondria. Then, the supernatants and precipitates were collected separately for subsequent assays as per the guidelines provided by the manufacturer. The overall mitochondrial complexes I‐V activity in the sample was the combined activity of complexs I‐V found in both the supernatants and the precipitates. All samples were extracted and tested on the same day. The final data were standardised based on sample quality.

### 4.11 Statistical Analysis

The mean ± standard error of mean (SEM) was used to present the experimental data. Student's *t*‐test and one‐way ANOVA were used to evaluate the differences between the data using GraphPad Prism software Version 6.01 (GraphPad Inc., La Jolla, CA, USA). *p < 0.05; **p < 0.01; ***p < 0.001.

## Author Contributions

J.Y., B.Z., C.S. and Z.G. initiated the project, designed the study, coordinated the experiment and wrote the manuscript. W.X., J.Y., Q.H., Y.Q., Q.H., C.S., X.C. and Q.X. performed the experiments and provided conceptual inputs for the paper. J.L., Z.P., Y.Z., G.H., J.H., Y.L., Z.C. and Y.Z. analysed the data. All authors read and approved the final manuscript.

## Ethics Statement

This study was approved by the Welfare Ethics Review Committee of the Experimental Animal Center of Nantong University.

## Conflicts of Interest

The authors declare no conflicts of interest.

## Supporting information


**Figure S1.** Heatmap representation of the top 5 highly expressed genes within each testicular cell cluster. Colour intensity reflects the average normalised expression level.
**Figure S2.** Pseudotime trajectory analysis of germ cells in testes aged 2‐days and 40‐days. (A) Ridge plot representation of the pseudotime trajectory for germ cell populations in the two age groups. (B) Density visualisation of the pseudotime trajectory for germ cell populations in 2‐days and 40‐days aged testes. (C) Pseudotime trajectory analysis of germ cells distinguished by various samples.
**Figure S3.** Analysis of DEGs between testes aged 2‐days and 40‐days. (A) Venn diagram analysis highlighting commonly down‐regulated DEGs across germ cell populations. (B) Venn diagram analysis illustrating commonly up‐regulated DEGs across germ cell populations. (C) Barplot representations showcasing GO enrichment related to mitochondrial‐associated events in each germ cell population under the category of cellular components. (D) Barplot views displaying GO enrichment related to mitochondrial‐associated events in each germ cell population under the category of biological processes. (E) UMAP visualisation of germ cells in testes aged 2‐Days and 40‐Days. Cell clusters are indicated by distinct colours.
**Figure S4.** Validations of mitochondria‐associated targets during testicular ageing. (A) RNA FISH analysis of Hsp60B (red) at 2‐Days and 40‐Days testes, with Vasa staining for the germline cell (green). (B) RNA FISH analysis of fzo (red) at 2‐Days and 40‐Days testes, with Vasa staining for the germline cell (green). (C) RNA FISH analysis of Tim17b1 (red) at 2‐Days and 40‐Days testes, with Vasa staining for the germline cell (green). (D) RNA FISH analysis of mRpL12 (red) at 2‐Days and 40‐Days testes, with Vasa staining for the germline cell (green). DNA was counterstained with Hoechst33342. Scale bar: 50 μm.
**Figure S5.** Examinations of mitochondrial respiratory chain activities during testicular ageing. (A) Mitochondrial complex I/NADH dehydrogenase activity in testes aged 2‐Days and 40‐Days. (B) Mitochondrial complex II/Succinate‐Coenzyme Q reductase activity in testes aged 2‐Days and 40‐Days. (C) Mitochondrial complex III/CoQ‐Cytochrome C reductase activity in testes aged 2‐Days and 40‐Days. (D) Mitochondrial Complex IV/Cytochrome C oxidase activity in testes aged 2‐Days and 40‐Days. (E) Mitochondrial Complex V/F1‐F0 ATP synthase activity in testes aged 2‐Days and 40‐Days. *p < 0.05, **p < 0.01, ***p < 0.001.

## Data Availability

The raw sequence data reported in this paper have been deposited in the Genome Sequence Archive (Genomics, Proteomics & Bioinformatics 2021) in National Genomics Data Center (*Nucleic Acids Res* 2022), China National Center for Bioinformation/Beijing Institute of Genomics, Chinese Academy of Sciences (CRA011800 and CRA008883) that are publicly accessible at https://ngdc.cncb.ac.cn/gsa.
