# Supplementary material for: Single‐Cell Transcriptomics Uncovers Core Signature for Regulating Mitochondrial Homeostasis During Testicular Ageing
Source: Cell Prolif. 2024 Dec 27;58(5):e13797. doi: 10.1111/cpr.13797 (PMC12099219; doi:10.1111/cpr.13797)
Supplement: Supplementary file 1 — Figure S1. Heatmap representation of the top 5 highly expressed genes within each testicular cell cluster. Colour intensity reflects the average normalised expression level. Figure S2. Pseudotime trajectory analysis of germ cells in testes aged 2‐days and 40‐days. (A) Ridge plot representation of the pseudotime trajectory for germ cell populations in the two age groups. (B) Density visualisation of the pseudotime trajectory for germ cell populations in 2‐days and 40‐days aged testes. (C) Pseudotime trajectory analysis of germ cells distinguished by various samples. Figure S3. Analysis of DEGs between testes aged 2‐days and 40‐days. (A) Venn diagram analysis highlighting commonly down‐regulated DEGs across germ cell populations. (B) Venn diagram analysis illustrating commonly up‐regulated DEGs across germ cell populations. (C) Barplot representations showcasing GO enrichment related to mitochondrial‐associated events in each germ cell population under the category of cellular components. (D) Barplot views displaying GO enrichment related to mitochondrial‐associated events in each germ cell population under the category of biological processes. (E) UMAP visualisation of germ cells in testes aged 2‐Days and 40‐Days. Cell clusters are indicated by distinct colours. Figure S4. Validations of mitochondria‐associated targets during testicular ageing. (A) RNA FISH analysis of Hsp60B (red) at 2‐Days and 40‐Days testes, with Vasa staining for the germline cell (green). (B) RNA FISH analysis of fzo (red) at 2‐Days and 40‐Days testes, with Vasa staining for the germline cell (green). (C) RNA FISH analysis of Tim17b1 (red) at 2‐Days and 40‐Days testes, with Vasa staining for the germline cell (green). (D) RNA FISH analysis of mRpL12 (red) at 2‐Days and 40‐Days testes, with Vasa staining for the germline cell (green). DNA was counterstained with Hoechst33342. Scale bar: 50 μm. Figure S5. Examinations of mitochondrial respiratory chain activities during testicular ageing. (A) Mito [file CPR-58-e13797-s001.docx]

**Supporting Information for**

**
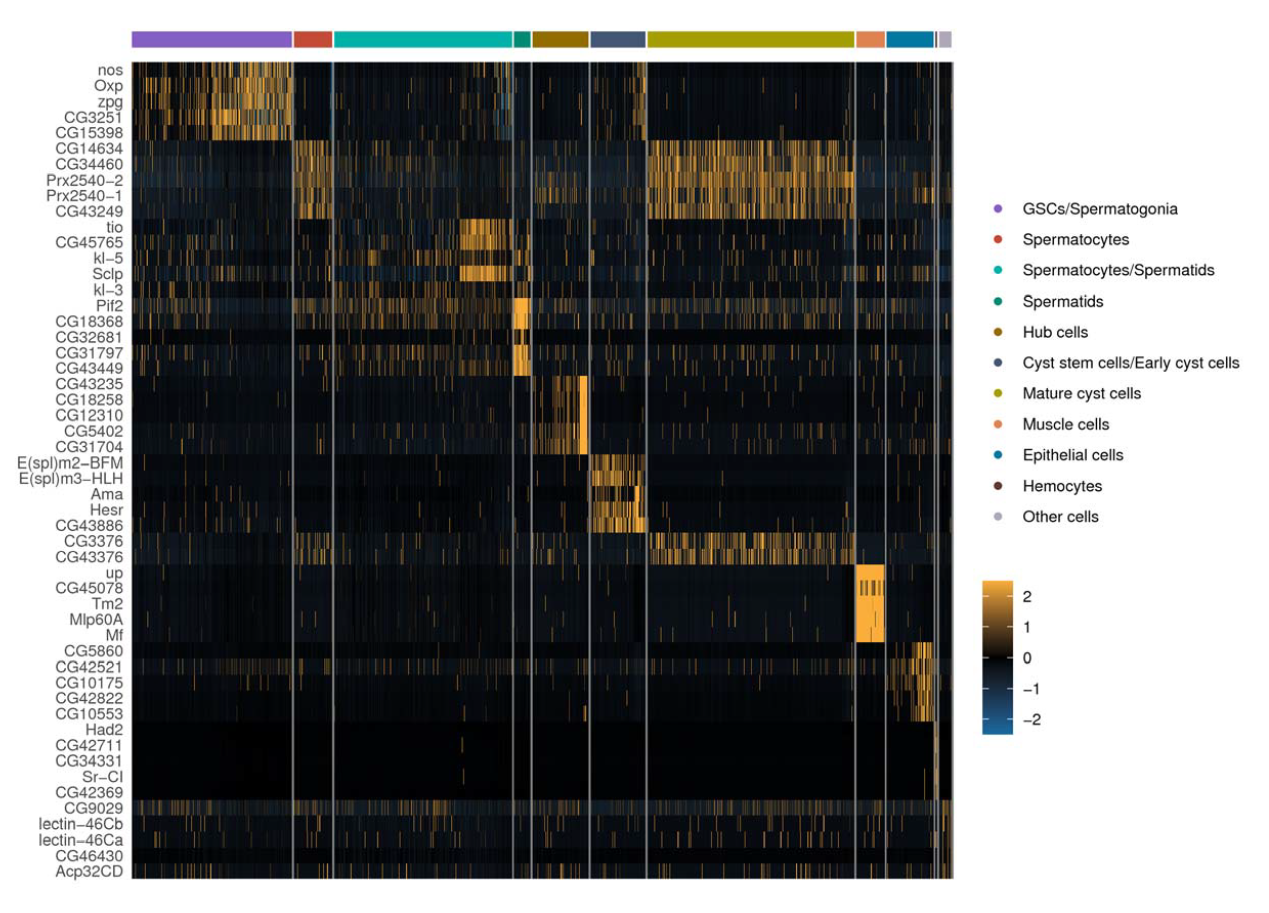
**

**Figure S1. Heatmap representation of the top 5 highly expressed genes within each testicular cell cluster.** Color intensity reflects the average normalized expression level.

**
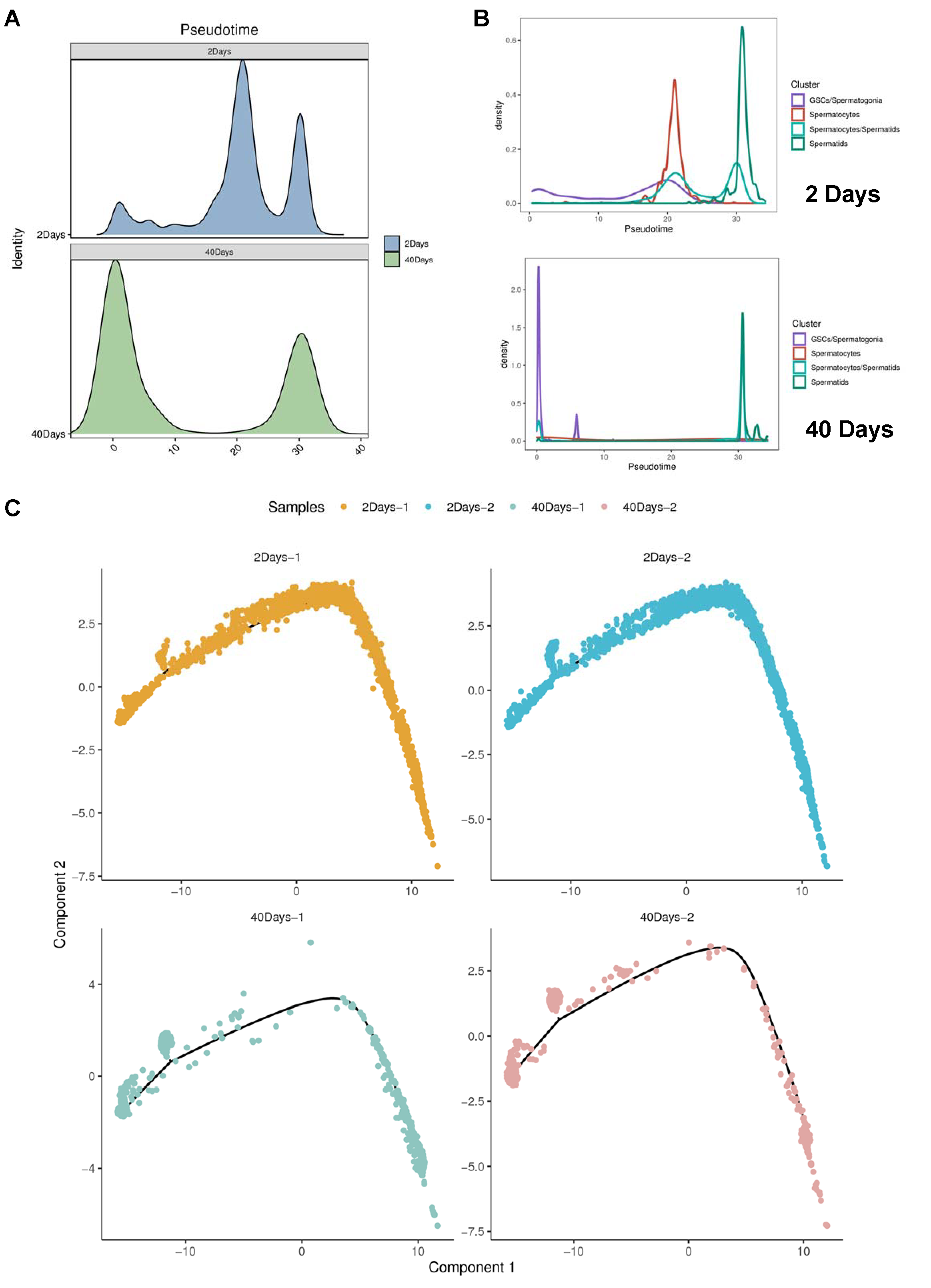
**

**Figure S2. Pseudotime trajectory analysis of germ cells in testes aged 2-Days and 40-Days.** (A) Ridge plot representation of the pseudotime trajectory for germ cell populations in the two age groups. (B) Density visualization of the pseudotime trajectory for germ cell populations in 2-Days and 40-Days aged testes. (C) Pseudotime trajectory analysis of germ cells distinguished by various samples.

**
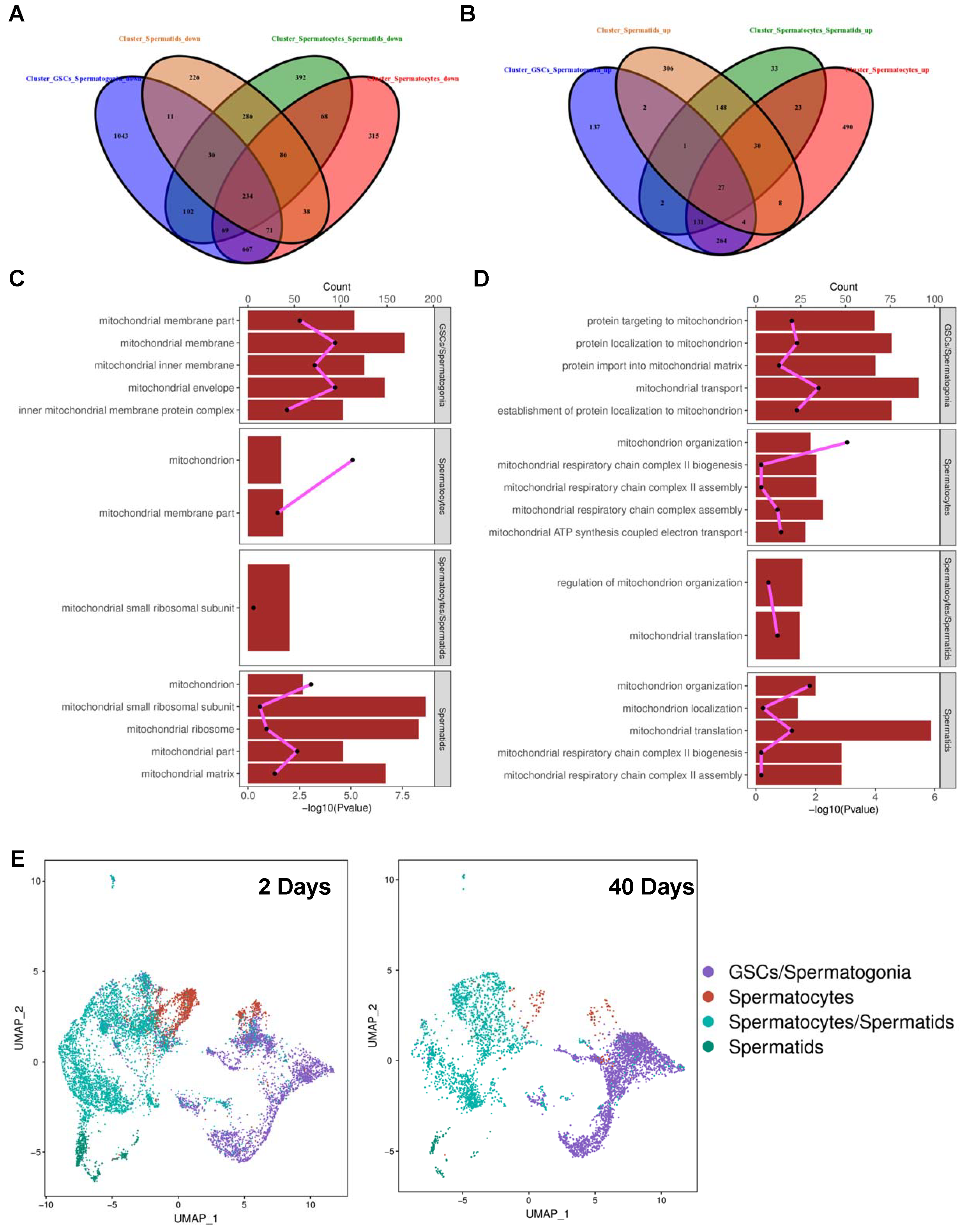
**

**Figure S3. Analysis of DEGs between testes aged 2-Days and 40-Days.** (A) Venn diagram analysis highlighting commonly down-regulated DEGs across germ cell populations. (B) Venn diagram analysis illustrating commonly up-regulated DEGs across germ cell populations. (C) Barplot representations showcasing GO enrichment related to mitochondrial-associated events in each germ cell population under the category of cellular components. (D) Barplot views displaying GO enrichment related to mitochondrial-associated events in each germ cell population under the category of biological processes. (E) UMAP visualization of germ cells in testes aged 2-Days and 40-Days. Cell clusters are indicated by distinct colors.

**
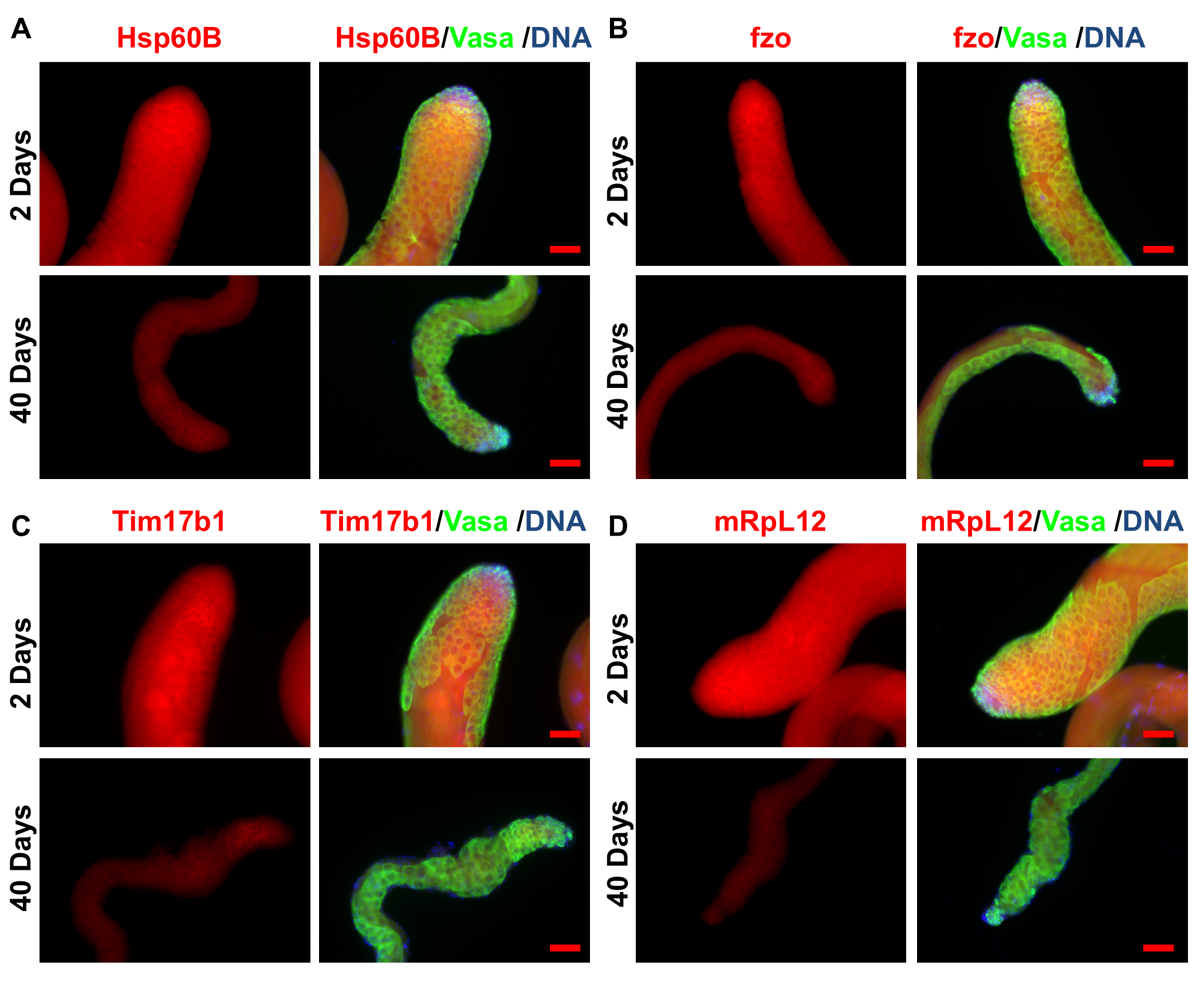
**

**Figure S4. Validations of mitochondria-associated targets during testicular aging.** (A) RNA FISH analysis of Hsp60B (red) at 2-Days and 40-Days testes, with Vasa staining for the germline cell (green). (B) RNA FISH analysis of fzo (red) at 2-Days and 40-Days testes, with Vasa staining for the germline cell (green). (C) RNA FISH analysis of Tim17b1 (red) at 2-Days and 40-Days testes, with Vasa staining for the germline cell (green). (D) RNA FISH analysis of mRpL12 (red) at 2-Days and 40-Days testes, with Vasa staining for the germline cell (green). DNA was counterstained with Hoechst33342. Scale bar: 50 μm.


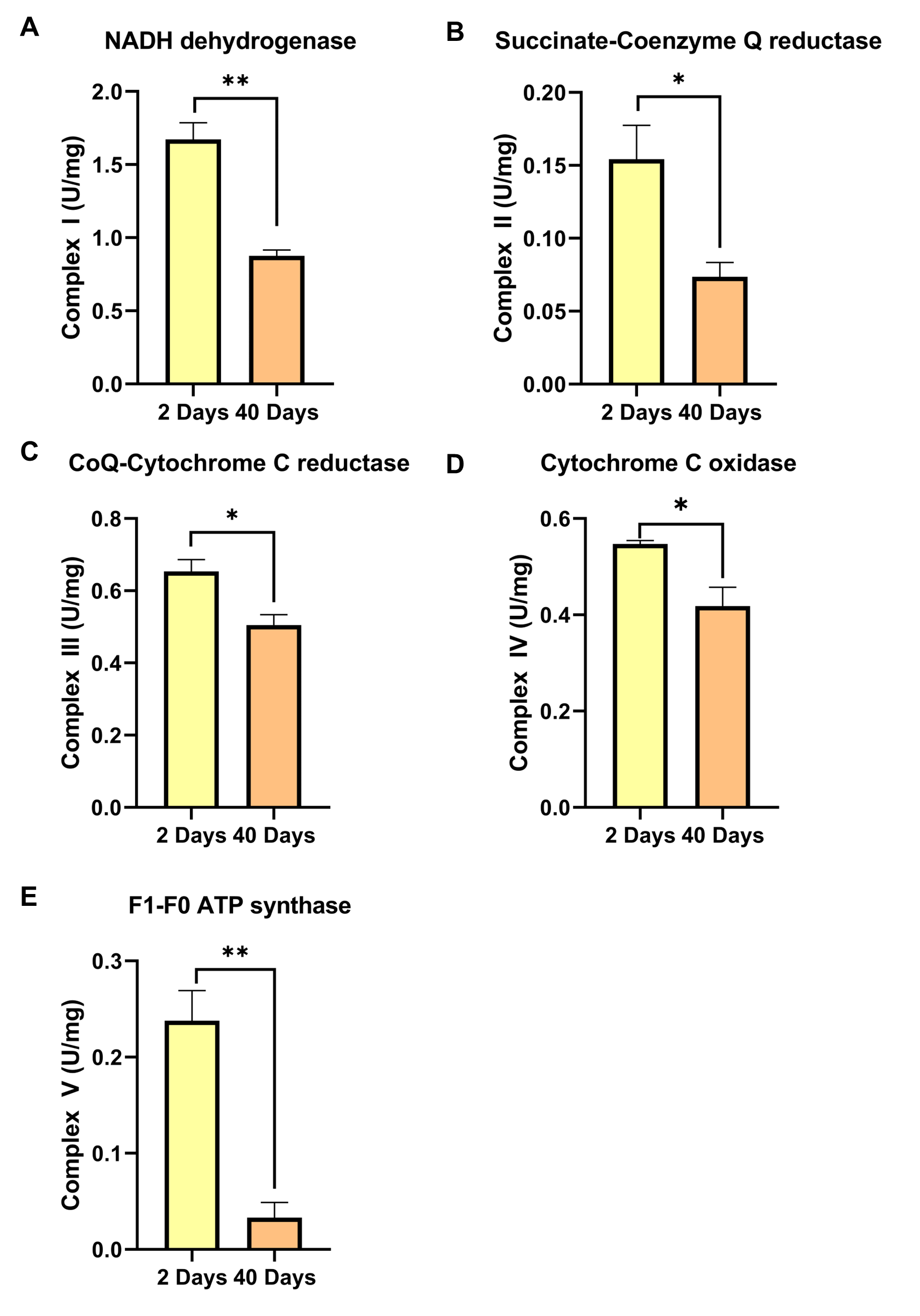


**Figure S5. Examinations of mitochondrial respiratory chain activities during testicular aging.** (A) Mitochondrial complex I/NADH dehydrogenase activity in testes aged 2-Days and 40-Days. (B) Mitochondrial complex II/Succinate-Coenzyme Q reductase activity in testes aged 2-Days and 40-Days. (C) Mitochondrial complex III/CoQ-Cytochrome C reductase activity in testes aged 2-Days and 40-Days. (D) Mitochondrial Complex IV/Cytochrome C oxidase activity in testes aged 2-Days and 40-Days. (E) Mitochondrial Complex Ⅴ/F1-F0 ATP synthase activity in testes aged 2-Days and 40-Days. * *P* <0.05, ** *P* <0.01, *** *P* <0.001.
